# Validation of the modified Alvarado score on patients attending A&E units with suspected appendicitis

**DOI:** 10.1186/s12873-023-00846-2

**Published:** 2023-08-10

**Authors:** Eszter Mán, András Szilágyi, Zsolt Simonka, Ferenc Rárosi, Zoltán Pető, György Lázár

**Affiliations:** 1 Department of Surgery, University of Szeged, 6720 Szeged, Semmelweis Street 8, Szeged, Hungary; 2 Department of Medical Physics and Informatics, University of Szeged, Szeged, Hungary; 3 Department of Emergency Medicine, University of Szeged, Szeged, Hungary

**Keywords:** Modified Alvarado score, Acute appendicitis, Emergency department, Validation, Ultrasound, Emergency residents

## Abstract

**Introduction:**

The aim of our prospective study was to confirm validity and diagnostic accuracy of the modified Alvarado score, which was developed at the Department of Surgery, University of Szeged, on patients presenting with symptoms suggestive of acute appendicitis (right lower quadrant complaints) at the A&E department.

**Patient population, methods:**

138 patients were included in our study between 01.01.2019 and 01.01.2020. For patients attending A&E, the first medic calculated and recorded the modified Alvarado score before surgical consultation. The consulting surgeon decided on further treatment without knowing the score. Validation of the score was based on the pathology report of the removed appendix (whether the operation was warranted, and if the score also supported indication for surgery), if there was readmission or surgery due to worsening symptoms after discharge from A&E. We also examined if there was any connection between the value of the Alvarado score and the severity of inflammation. Our aim was to prove that using modified Alvarado score at the A&E Units helps to reduce patient’s waiting time and avoid unnecessary surgical consultations. Furthermore our study included measuring the diagnostic accuracy of the ultrasound examination (specificity, sensitivity).

**Results:**

Based on the results, patients presenting at A&E had a mean modified Alvarado score of 6.5. Comparing the score to histological results showed that the specificity of the modified Alvarado score was 100%, and its sensitivity was 80.7%. Based on Spearman’s rank correlation (0.796) and ROC analysis (AUC 0.968), the modified Alvarado score has an excellent predictive value in diagnosing acute appendicitis. When comparing the patients’ waiting times with the use of modified Alvarado score and without it we found that there was a significant difference in group also in group under 4 points and in group over 7 points when using modified Alvarado score, so the diagnostic and therapeutic algorithm should be much quicker with the help of the score. We found a correlation between the severity of inflammation based on the Fisher’s exact test. Rank correlation of the same question also showed a significant connection. All patients had an US examination during their diagnostic course, its sensitivity was 82.6%, specificity was 87%. Based on this, we can conclude that the predictive value of the imaging method is good.

**Conclusions:**

We can conclude according to our results that the predictive value of the modified score is excellent, and it can be safely applied by non-surgeons in urgent care in the differential diagnosis of acute appendicitis. The new score incorporates the results of an easily obtainable, ionising radiation free imaging method, the ultrasound, which was not included in previous scores. With the help of the new score, the number of unnecessary surgical referrals and waiting times for patients are reduced, excess examinations will become avoidable.

## Introduction

Acute appendicitis is the most common surgical emergency. Presently, its incidence in Europe and the US is approximately 100 cases/100,000 people yearly [1]. The rate of appendicectomy in Hungary is 100 people/1,000,000 inhabitants per year, out of which 30/1,000,000 is laparoscopic appendectomy [2]. Based on a 0.7% mortality, approximately 100 people die of the disease every year in Hungary [3].

There was a paradigm change in diagnosis and treatment of appendicitis in recent years: now the question is not whether to use laparoscopy or an open technique as the surgical approach, but to even operate on early uncomplicated cases. There are also more questions arising in diagnostic management from choosing the appropriate imaging modalities to efficacy of scores. Two consensus conferences were organized in the past 6 years about acute appendicitis, highlighting the importance of this well-known, common disease. The first was organized by the World Society of Emergency Surgery (WSES) [4] in 2015, held in Jerusalem, then another concensus conference in 2020 to debate newly arising questions [5].

In the past years, different score systems were devised to facilitate diagnosis. The most widely known score system is the Alvarado score, which was designed by Alvarado in 1986, retrospectively examining data of post-appendectomy patients. The score system incorporates nine diagnostic criteria and further decisions on treatment are based on the overall score [6]. In addition, the Pediatric Appendicitis Score, Appendicitis Inflammatory Response Score (AIR), Raja Isteri Pengiran Anak Saleha Appendicitis Score (RIPASA) and Adult Appendicitis Score (AAS) should be noted. According to the summary of the results of studies validating the most common score systems by Kularatna et al., AIR has the greatest diagnostic accuracy, its sensitivity and specificity being 92% and 63%, respectively [7], but according to a prospective study in 2020 made by Elsherbiny et al., Alvarado score is specific in the diagnosis of acute appendicitis and could identify all patients with normal appendix [8]. Furthermore a systematic review by Gupta et al. suggests that high Alvarado score (7 and above) is a significant predictor for acute appendicitis [9].

We conducted a prospective, randomised study of comparing the diagnostic accuracy of Alvarado score to the traditional clinician’s decision approach at the Department of Surgery, University of Szeged between 2011.09.01 and 2012.12.31 (Impact of the Alvarado score on the diagnosis of acute appendicitis: comparing clinical judgement, Alvarado score and a new modified score in suspected appendicitis: a prospective, randomised clinical trial), which can help professionals working in urgent and emergency care in differential diagnosis of the disease. Ethics approval number: 248/2018-SZTE [10].

Based on our former study, diagnostic accuracy of the Alvarado score was lower than the accuracy of clinician’s decision, we aimed to weigh the parameters (using linear regression) to devise a new, more sensitive modified score system. We chose parameters that were not part of the score system before, but we considered them important based on our clinical experience and were statistically significant in diagnosing acute appendicitis (result of an ulstrasound scan) and also excluded parameters that were less significant depending on linear regression (e.g. rectal-axillary temperature difference). The diagnostic accuracy of the derived modified Alvarado score was increased [10] (Table 1).

**Table 1:** Modified Alvarado score Discharge: 1–4 points, admission and monitoring: 5–6 points, urgent operation: >7 points

	Score value
Nausea and vomiting	2
Right lower abdominal tenderness	2
Positivity of indirect signs (Blumberg, Rovsing, obturator, psoas sign) (1–2)	1
Positivity of indirect signs (Blumberg, Rovsing, obturator, psoas sign) (≥ 2)	2
Leucocytosis > 10 g/L	1
Leucocytosis > 15 g/L	2
US examination	2

The aim of our prospective study was to validate the score on patients presenting with right lower abdominal pain and confirm that the modified Alvarado score is a reliable tool for the non surgeon specialists in the differential diagnosis of suspected acute appendicitis cases. After validation, the modified Alvarado score can find its place in the daily operation of the A&E department in the case of patients with right lower abdominal pain. Unnecessary surgical referrals could be avoided with its use and the waiting times for patients for whom a surgical consultation is not justified could be reduced. On the other hand, in case of a high score, surgical admission could be quicker, shortening the time until the operation.

## Materials and methods

We aimed to validate the modified Alvarado score in our study between 2019.01.01 and 2020.01.01 at the Emergency Patient Care Unit of the University of Szeged with patients > 18 years presenting with right lower abdominal pain suggestive of acute appendicitis.

Modified Alvarado score was calculated and recorded by the attending accident and emergency (A&E) resident doctor before surgical referral for patients presenting at A&E. The consulting surgeon could not know this score, they decided on further treatment independently. Validating the score system was done by comparing the final pathology report – if there was surgical intervention – with the score (whether surgery was truly justified and if it was also indicated based on the score) or if there was readmission or surgical intervention due to worsening of symptoms if discharged from A&E.

We also examined if there was any connection between the value of the Alvarado score and the severity of inflammation. Our study also looked at the diagnostic accuracy of the ultrasound examination (specificity, sensitivity).

We investigated the effect of the Alvarado score on the length of patient’s waiting time and unnecessary surgical consultations. We recorded the time the patinent arrived at the A&E Unit and the time when the Alvarado score was calculated (after physical examination, laboratory test and ultrasound investigation). The difference between these two times was the SCORE TIME- ScT. After calculating the score surgical consultation was asked. We also recorded the waiting time from the score calculation until the surgical examination: CONSULTATION TIME: CoT. These two times gave the total waiting time for each patient: ScT + CoT = ToT. In patients group of modified Alvarado score less than 4 (Group 1, n = 60.), based on our former investigations, surgical consultation is not necessary as appendicitis is unlikely, so the CoT should be avoided. Ont he other hand, for patients with an Alvarado score 7 or more (Group 2, n = 50), as there’s a high risk for appendicitis, there’s no need for an extra surgical consultation. Patients shold be admitted to a surgical ward. So in this patient’s group CoT is unnecessary again. We investigated if there’s a significant difference between ScT and ToT in both Group 1 and 2. To prove the effect of modified Alvarado score on patient’s waiting time we calculated the difference between ScT and the TOTAL WAITING TIME (ToT) of the patients with 4 or less points (Group 1) and with 7 or more points (Group 2) (ToT = ScT + CoT) with paired sample test, to see if there’s a significant difference in waiting times with and without the use of modified Alvarado score.

We calculated specificity and sensitivity for the ultrasound examination with comparing to the final pathology report by using crosstabulation. We performed ROC curve analysis to analyse the diagnostic accuracy of the modified Alvarado score. Statistical calculations were performed with IBM SPSS 26 software. Level of significance was p < 0.05.

## Results

**Fig. 1:**
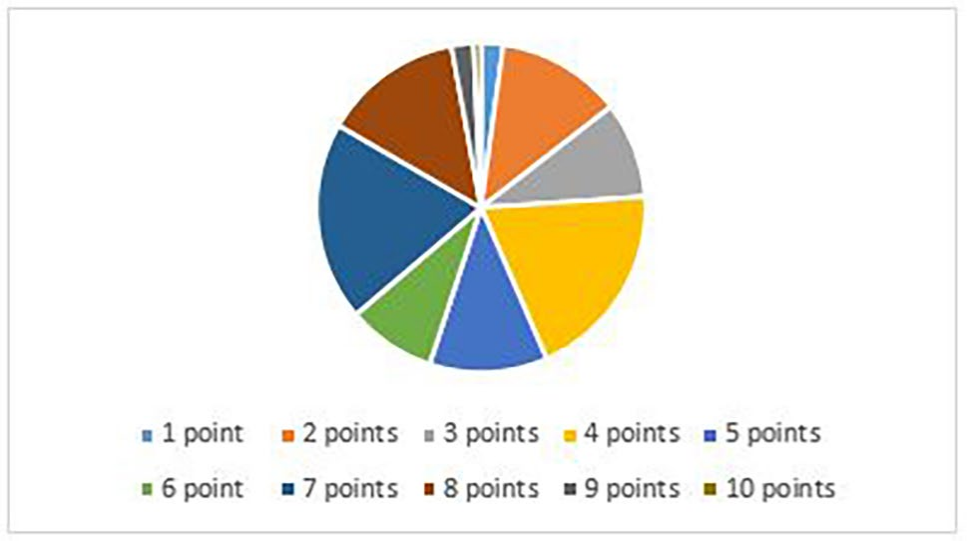
Alvarado scores (n = 138)

138 patients were included in our study between 01.01.2019 and 01.01.2020. Mean age of patients were 32 years (18–67). The number of females was 93, the number of males was 45. The mean modified Alvarado score was 6.5: 1-n = 3, 2-n = 17, 3-n = 13, 4-n = 27, 5-n = 16, 6-n = 12, 7-n = 27, 8-n = 19, 9-n = 3, 10-n = 1. The main groups were: 1–4 points (discharge) n = 60, 5–6 points (observation) n = 28, 7–10 points (urgent surgery) n = 50 (Fig. 1).

Diagnostic value of the modified Alvarado score in diagnosing acute appendicitis: we have compared final pathology reports with the modified Alvarado score of the patients (group 0 – no acute appendicitis, either no surgery performed or negative pathology report n = 87), group 1 – other pathology was confirmed (cancer, diverticulum) (n = 5), group 2 – mild inflammation (simple, phlegmonous, superficial acute appendicitis) (n = 20), group 3 – severe inflammation (ulcero-phlegmonous, gangrenous acute appendicitis, perforation) (n = 26). Compared to the pathology results, the specificity and sensitivity of the modified Alvarado score was 100% and 80.7%, respectively.

**Fig. 2:**
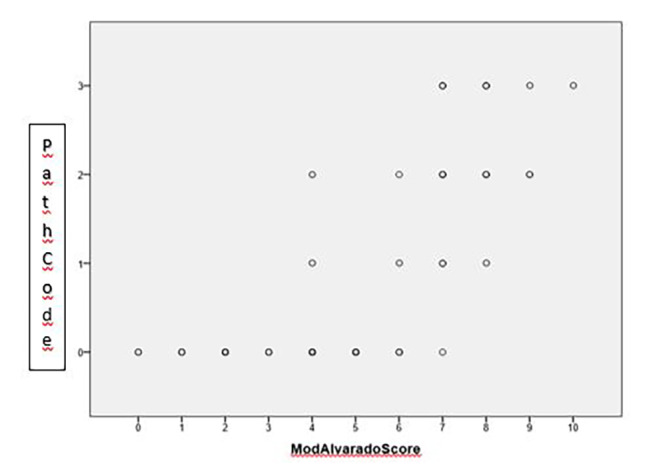
Correlation of the Modified Alvarado score and the severity of inflammation (histology result) based on Spearman’s rank correlation

**Fig. 3:**
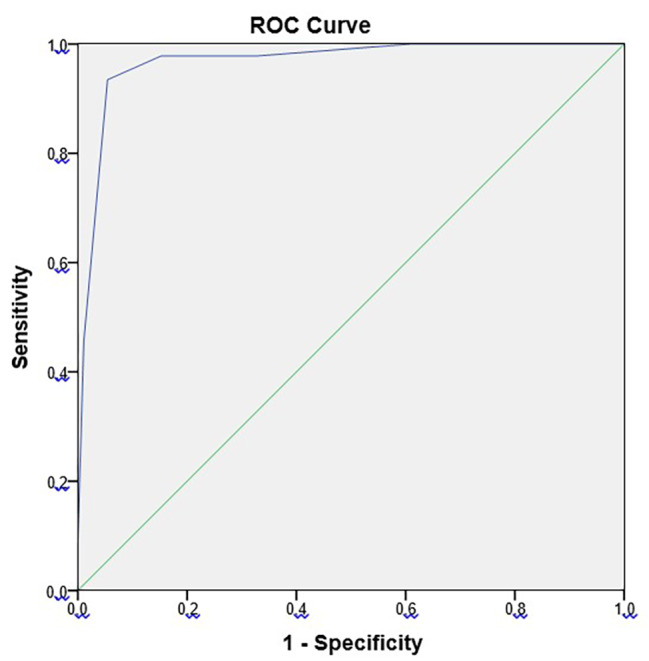
ROC curve analysis of the modified Alvarado score and the severity of inflammation (histology result)

Based on the above, we can conclude that correlation between the modified Alvarado score and the final pathology result was fairly close: 0.796 and significant, too (Fig. 2). There was no negative pathology result (group 0) above a score of 4. Coding pathology results as 0 and 1 being 0 (i.e. no appendicitis), and 2 and 3 as 1 (i.e. appendicitis) yields an AUC of 0.968 on ROC curve analysis, which means an excellent distinction (Fig. 3).

**Fig. 4:**
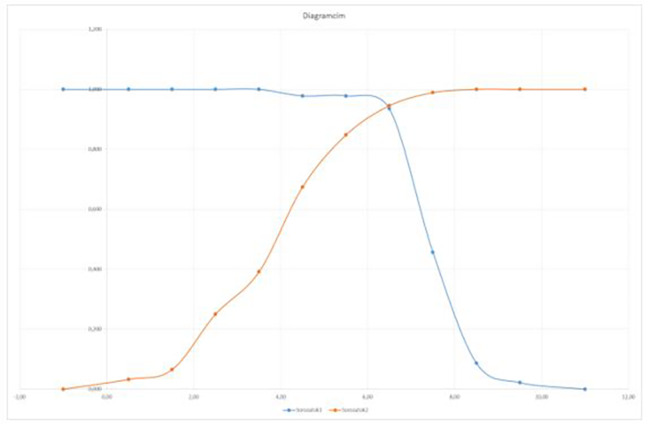
Modified Alvarado score and histological code cross tabulation – Youden index

The question is raised if the two distinction points given for the original Alvarado score – 4 points (probability of acute appendicitis is low) and 7 points (surgery is indicated as the probability of acute appendicitis is high) and the grey zone 5–6 points when further observation and imaging (CT) is needed – is also valid for our modified Alvarado score. We can conclude that the modified Alvarado score also has the same distinction points, that is below 4 points probability of appendicitis is low and above 7 points it is high. For patients with scores of 5–6 points, further observation, recalculation of score and further imaging (urgent CT scan) is recommended (Table 2; Fig. 4).

**Table 2:** Modified Alvarado score and pathology code cross tabulation

		Pathology code				
		0	1	2	3	Total
Modified Alvarado score	0	3	0	0	0	3
	1	3	0	0	0	3
	2	17	0	0	0	17
	3	13	0	0	0	13
	4	25	1	1	0	27
	**5**	**16**	**0**	**0**	**0**	**16**
	**6**	**8**	**1**	**2**	**0**	**11**
	7	2	2	8	14	26
	8	0	1	7	10	18
	9	0	0	2	1	3
	10	0	0	0	1	1
Total		87	5	20	26	138

Pathology code:

group 0 – no acute appendicitis, either no surgery performed or negative pathology,

group 1 – other pathology was confirmed (cancer, diverticulum)

group 2 – mild inflammation (simple, phlegmonous, superficial acute appendicitis),

group 3 – severe inflammation (ulcero-phlegmonous, gangrenous acute appendicitis, perforation)

78 patients were discharged from A&E based on clinician’s decision, all of these patients had an Alvarado score less than 4. There was one case when there was a repeat surgical referral after 24 h and the consulting surgeon decided on operating (repeat Alvarado score: 7 points). 60 patients had an operation: 56 of which had a laparoscopic appendectomy, 1 had adhaesiolysis, 1 had right hemicolectomy, 1 had a Hartmann’s procedure due to sigmoid diverticulitis, and 1 had exploration only. Out of the 56 laparoscopic appendectomy cases, a drain was left behind in 23 cases. There was no conversion to laparotomy. The base of the appendix was closed using clips in 55 cases, while stapler was used in one case due to severity of inflammation.

The mean ScT was 194.83 min in Group 1 (= 60.) and 208.45 min in Group 2 (n = 50). The mean ToT was: 279.8276 min in Group 1 and 300,0909 min in Group 2. In both groups the difference between the ScT and ToT calculated with paired samples test was statistically significant (p < 0.01) (Table 3).

**Table 3:** Paired T test for waiting times in Group 1 and 2 (ScT- Score recording tome, ToT- total waiting time) (p < 0.05)

	Paired … 95% Confidence Interval of the … Upper			
		t	df	Sig. (2-tailed)
Group 1 Difference between ScT and ToT	-80,54680	-38,222	57	,000
	Paired … 95% Confidence Interval of the … Upper			
		t	df	Sig. (2-tailed)
Group 2 Difference between ScT and ToT	-85,46922	-29,790	54	,000

US examination was performed in all cases, because it is an item in the modified Alvarado score: No acute appendicitis was confirmed in 88 cases, while in 50 cases, US confirmed acute appendicitis. Supplementary CT scan was performed in 11 cases (7.9%), depending on the decision of the consulting surgeon. All patients had a negative US examination, inflamed appendix was not visualized: in 6 cases negative abdominal status was found, 3 patients had minimal free abdominal fluid and ileo-coecal wall thickening was proven in 2 cases, 9 patients had a modified Alvarado score of 5–6, 2 had less than 4 points. The CT scan confirmed the presence of appendicitis in 7 cases, it was negative in 3 cases, while an intestinal conglomerate was reported in the right lower abdomen with abscess formation in 1 case.

By comparing the results of the ultrasound examination with final histology reports, we calculated the diagnostic accuracy of the imaging study: sensitivity was 82.6%, specificity was 87%. We can conclude that the predictive value of the imaging method is good (Table 4).

**Table 4:** Cross tabulation of US and histology results, specificity and sensitivity of ultrasound

			Histology result		Total
			0.00	1.00	
US result	0.0	Count	80	8	88
		% within Histology	87.0%	17.4%	63.8%
	1.0	Count	12	38	50
		% within Histology	13.0%	82.6%	36.2%
Total		Count	92	46	138
		% within Histology	100.0%	100.0%	100.0%

## Discussion

Our previous prospective study (Impact of the Alvarado score on the diagnosis of acute appendicitis: comparing clinical judgement, Alvarado score and a new modified score in suspected appendicitis: a prospective, randomised clinical trial) confirmed the diagnostic utility of the Alvarado score, which can help professionals working in urgent and emergency care in differential diagnosis of the disease: Specificity of Alvarado score (group A) in our former study was 88.9%, while the specificity of traditional clinician’s decision approach (group B) was 94.8% (p = 0.320). The rate of histologically negative appendicectomies was 8.42% in group A and 3.62% in group B (p = 0,160). It can be concluded that the diagnostic accuracy of Alvarado score is good, but it is inferior to the traditional, surgeon’s decision approach. However, it is an excellent diagnostic tool in A&E to facilitate choosing patient pathway (surgical referral, further imaging, discharge etc.). Arzu et al. compared the diagnostic accuracy of emergency medicine trainees using Alvarado score with the predictive value of surgical trainees not using the score in a prospective study. Sensitivity of the Alvarado score was 95.4%, specificity was 45.7%. There was no significant difference between the positive and negative predictive values of emergency medicine trainees using the score and surgical trainees not using it [11]. In another prospective study, You et al. compared the predictive value of surgical trainees, emergency medicine trainees, Alvarado score and CT abdomen. The abdominal CT scan had the highest diagnostic accuracy, followed by Alvarado score. The least predictive was trainees’ decision, but there was no significant difference between the diagnostic accuracy of surgical and non-surgical trainees [12]. Ünlüer et al. also investigated the predictive value of emergency physicians, but they also involved a new element into their study, a bedside ultrasound (BUS) performed by emergency doctors or radiologists. They found that BUS performed by emergency doctors is just moderately useful in detecting appendicitis, but when it’s combined with Alvarado score, it’s a perfect for ruling out appendicitis in emergency departments [13].

The 2020 WSES guideline states that a score of less than the cutoff of Alvarado score, i.e. 5 points, can rule out acute appendicitis with an acceptable sensitivity, but its real strength is that waiting times at the A&E department can be reduced, unnecessary imaging studies and surgical referrals could be avoided with the use of the score.

We concluded via statistical analysis that by including the result of the ultrasound scan and dropping certain parameters and weighing some others, our modified Alvarado score could increase diagnostic accuracy. The aim of our study was to validate the modified score in practice.

The prospective clinical trial involving 138 patients with right lower abdominal pain confirmed that having an ultrasound scan in every case has a high sensitivity (82.6%) and specificity (87%). Due to its good predictive value, it has to be included among the criteria of the Alvarado score, as it can support more accurate diagnosis of the disease. These results correlate well with international data, showing ultrasound has a specificity of 95% and sensitivity of 76% [14]. A review from Hang et al. suggests that ultrasound is an effective first-line tool in the diagnosis of acute appendicitis with a sensitivity of 86%, specificity of 94%, positive predictive value 100% and negative predictive value 92% [15]. A study by Altomare et al. also recommends ultrasound as a first line instrumental examination, especially in the exclusion of low risk patients [16].

In our study, the mean modified Alvarado score was 6.5. 79 patients were discharged from A&E based on clinician’s decision, all of these patients had an Alvarado score less than 4. There was one case (1.3%) with readmission and appendectomy, but in that case (compared to the first score of less than 4), the repeat Alvarado score was increased (7 points).

It is very important to decide what to do with the cutoff points of the original Alvarado score: 5 points (below which probability for appendicitis is low) and 7 points (appendicitis has a high probability – surgery advised). Would these be valid for the modified Alvarado score and what should be the treatment plan for those in the grey zone (5–6 points) (observation, further imaging). There was a study in 2011 exploring these cutoff points of the original Alvarado score. The cutoff point at 5 points had a sensitivity of 99% for excluding appendicitis. A cutoff point between 7 and 10 points had a sensitivity of 82% for the presence of appendicitis [17]. There was a further study exploring the question of including the results of a CT scan in the original score, due to the fact that points 5–6 only have a sensitivity of 35.6% for confirming the disease, while the CT scan had a sensitivity of 90.4% in the population studied [18]. Overall, between 0 and 3 points, a CT scan is pointless, because the possibility of appendicitis is minimal, between 7 and 10 points, surgical consultation is warranted. Patients having a score of 4–6 points or rather 5–6 are possible candidates for a CT scan. It has to be observed though that a CT scan confirms radiation exposure, it has a high cost and it can lengthen time to surgery considerably on certain occasions. A retrospective study from Jones et al. showed that those patients who have Alvarado score less than 3 points do not benefit from CT investigation as CT showed no appendicitis or other findings in patients with Alvarado < 3 points [19]. Another retrospective study with 300 patients by Reddy et al. showed that a combined ultrasound and Alvarado score can replace the need for CT with a specificity of 82%, sensitivity of 98% at 6.5 Alvarado points and 87% specificity and 95% sensitivity at the score of 7.5 [20]. One retrospective and one prospective analysis from Tan WJ et al. proved that CT is beneficial mainly in patients with an Alvarado score between 4 and 8 points [21, 22]. A randomized controlled study by Noori et al. with 286 included patients found that CT scan is beneficial only for patients with equivocal clinical scores, in case of high scores surgery is needed. For patients with low points, where acute appendicitis is unlikely, ultrasound is enough to exclude other diagnoses [23]. Altough nowadays guidelines provide evidence based recommendations for the diagnostic and therapeutic steps for acute appendicitis for clinicans, everyday practice doesn’t always follow these guidelines. A prospective obsertvational cohort study from Bass et al. investigated the practice in diagnosis and management of appendicitis in 71 centers worldwide, if there’s a deviation from WSES Jerusalem guidelines for the diagnosis and management of acute appendicitis. They found that everyday practice wasn’t congruent with recommendations in the choice of diagnostic modalities and further investigations are needed to close the evidence-to-practice gap [24]. The US scan however should be included in the score as an individual criterion. There is no radiation exposure, it is easy to carry out, cheap, can be repeated and has an excellent predictive value if performed by an experienced radiologist. Though this modality also has some disadvantages: visualization is challenging in obese patients and due to the variability of investigating radiologists, evaluation is not objective. Since US should be the first line imaging modality in acute appendicitis [25].

We performed surgery for 60 patients in total: 56 of these were laparoscopic appendectomies, 4 patients had a different procedure due to the intraoperative findings.

Clinical score systems alone can help differentiate low risk patients, for whom the probability of having acute appendicitis is low. According to Andersson et al. the use of the AIR (Appendicitis Inflammatory Response) score led to significantly less imaging studies, unnecessary surgical admissions and negative surgical explorations [26]. The Adult Appendicitis Score (AAS) showed similar results, with grouping patients into three categories: low, moderate and high risk. In ROC curve analyis, its diagnostic accuracy was significantly higher than that of the Alvarado score or the AIR score [27]. A prospective study from Kollar et al. found that AIR score and Alvarado score are reliable in excluding appendicitis in low risk patients and in predicting appendicitis in high risk patients, in case of medium risk patients CT investigation should be considered [28].

We found a rather close correlation between our modified Alvarado score and the final histology result using Spearman’s rank correlation (0.796). ROC curve analysis showed an area under the curve of 0.968, which denotes an excellent cutoff value. Our prospective study confirmed that the modified Alvarado score has an excellent predictive value in recognizing acute appendicitis; its advantage being that it incorporates the results of the imaging method of first choice, ultrasound.

We also aimed to prove that the modified score can shorten patient’s waiting time, used by emergency doctors patients with a score 4 or less or 7 or more can benefit the most from the scoring system. They don’t have to wait for surgical consultation, they can be emitted or admitted to the surgical ward after score calculation. Still patients with 5 and 6 points can still have further imaging investigations (CT scan) and surgical consultation. Coleman et al. in their retrospective study also showed that CT scan and elevated waiting time due to the imaging technique is unnecessary in patients with low (1–2 points) or high (9–10) Alvarado points [29]. We found significantly shorter waiting times the use of Alvarado score compared to the total waiting time, including surgical consultation time as well. Of course in the so called ”grey zone”, 5–6 points, we also suggest that further imaging technique and surgical examination is needed before decision making. Furthermore longer waiting time and the delay of surgery can lead to complications as well. A study including 2136 patients by Andert et al. showed that for the subgroup of patients with complicated appendicitis, the time interval to surgery had a significant influence on the occurrence of postoperative complications [30]. Another study with more than 4000 included patients proved that delay in surgery meant a significantly higher risk for SSI with non-perforated appendicitis [31]. According to a study of Quevedo-Fernandez et al patients with more than 12 h deferral time to surgery had a more complicated clinical presentation,, higher frequency of abscess formation, higher need for surgical drainage and a longer hospital stay [32]. Overall we can suggest that both patients and health care systems, hospitals can benefit from shorter waiting times as we can aviod additional morbidities which can lead to longer hospital stay, further complications and additional financial costs.

## Conclusions

The predictive value of the new, modified score system designed at the Department of Surgery, University of Szeged is excellent and can safely be used in the case of right lower abdominal pain for diagnosis and differential diagnosis of acute appendicitis. It is important to note that contrary to other score systems it incorporates an imaging modality, ultrasound, which is quick, cheap and easy to acquire and has a good predictive value in diagnosing acute appendicitis according to international publications. It can be a helpful tool for non-surgeon doctors/specialists. Patient pathways at A&Es can be streamlined, the number of unnecessary surgical referrals and the waiting times for patients could be reduced with its daily use.

## Data Availability

The datasets used and/or analysed during the current study are available from the corresponding author on reasonable request.

## List of abbrevations

A&E: Department: accident and emergency department
AAS: Adult Appendicitis Score
AIR: Appendicitis Inflammatory Response Score
AUC: Area under the curve
BUS: Bedside ultrasound
CoT: Consultation time
CT: Computer tomography
RIPASA: Raja Isteri Pengiran Anak Saleha Appendicitis Score
ROC: Receiver operating characteristics
ScT: Score Time
ToT: Total waiting time
US: Ultrasound
WSES: World Society of Emergency Surgery
